# Peruvian Mental Health Reform: A Framework for Scaling-up Mental Health Services

**DOI:** 10.15171/ijhpm.2017.07

**Published:** 2017-01-22

**Authors:** Mauricio Toyama, Humberto Castillo, Jerome T. Galea, Lena R. Brandt, María Mendoza, Vanessa Herrera, Martha Mitrani, Yuri Cutipé, Victoria Cavero, Francisco Diez-Canseco, J. Jaime Miranda

**Affiliations:** ^1^ CRONICAS Center of Excellence in Chronic Diseases, Universidad Peruana Cayetano Heredia, Lima, Peru.; ^2^ Instituto Nacional de Salud Mental “Honorio Delgado - Hideyo Noguchi,” Lima, Peru.; ^3^ Socios en Salud, Lima, Peru.; ^4^ Department of Global Health and Social Medicine, Harvard Medical School, Boston, MA, USA.; ^5^ Ministerio de Salud, Lima, Peru.; ^6^ School of Medicine, Universidad Peruana Cayetano Heredia, Lima, Peru.

**Keywords:** Policy Analysis, Mental Health Services, Low- and Middle-Income, Health Systems, Healthcare Reform

## Abstract

**Background:** Mental, neurological, and substance (MNS) use disorders are a leading cause of disability worldwide; specifically in Peru, MNS affect 1 in 5 persons. However, the great majority of people suffering from these disorders do not access care, thereby making necessary the improvement of existing conditions including a major rearranging of current health system structures beyond care delivery strategies. This paper reviews and examines recent developments in mental health policies in Peru, presenting an overview of the initiatives currently being introduced and the main implementation challenges they face.

**
Methods:** Key documents issued by Peruvian governmental entities regarding mental health were reviewed to identify and describe the path that led to the beginning of the reform; how the ongoing reform is taking place; and, the plan and scope for scale-up.

**Results:** Since 2004, mental health has gained importance in policies and regulations, resulting in the promotion of a mental health reform within the national healthcare system. These efforts crystallized in 2012 with the passing of Law 29889 which introduced several changes to the delivery of mental healthcare, including a restructuring of mental health service delivery to occur at the primary and secondary care levels and the introduction of supporting services to aid in patient recovery and reintegration into society. In addition, a performance-based budget was approved to guarantee the implementation of these changes. Some of the main challenges faced by this reform are related to the diversity of the implementation settings, eg, isolated rural areas, and the limitations of the existing specialized mental health institutes to substantially grow in parallel to the scaling-up efforts in order to be able to provide training and clinical support to every region of Peru.

**Conclusion:** Although the true success of the mental healthcare reform will be determined in the coming years, thus far, Peru has achieved a number of legal, policy and fiscal milestones, thereby presenting a unique and fertile environment for the expansion of mental health services

## Background

Over the last 12 years, Peru has achieved certain milestones in the legal, policy, and fiscal fields. These milestones present a unique and fertile environment for mental health, laying the ground for the current mental health reform that is underway in the Peruvian public health system. This paper describes the panorama of mental health disorders and care in Peru and the public health system’s response; reviews key policy documents driving the mental health reform initiatives being implemented; and, puts forth potential challenges to be faced in this process.******The ongoing reform in Peru points towards a major (re)arranging of the health sector to accommodate a platform that is able to support mental health-related initiatives. As with many countries in transition, the public sector and health-related actors operate with concurrent and multiple competing demands and deficiencies. This review centers on the foundational landmarks achieved for mental health in Peru, primarily legal and budgetary in nature, and aims to serve as a reference point for future analyses of the effectiveness of the mental healthcare reform in Peru.

### Worldwide prevalence of mental disorders

Worldwide, mental, neurological, and substance (MNS) use disorders account for 9 out of the 20 leading causes of years lived with disability and 10% of the global burden of disease^1^; more than 80% are in low- and middle-income countries (LMICs). Indeed, by 2030 depression is projected to be the leading cause of disease burden, surpassing heart disease, injuries, and HIV/AIDS.^2^ Despite this burden, health systems have not adequately responded in kind, resulting in an enormous treatment gap (ie, the difference between the number of people requiring treatment and those who receive treatment) that must be addressed.^3^ A review of the global literature found MNS treatment gaps to be very high,^4^ and in LMIC, between 76%-85% of people with severe mental disorders receive no treatment.^3-5^ Most LMIC have few mental health human resources available, and those that exist are inadequately trained or are inefficiently distributed within the health system^4^ leading to ineffective or inappropriate treatment and a low probability of recovery.^2^

Because of these issues, mental health has reached the international agenda, forging the global mental health movement. The Grand Challenges in Global Mental Health initiative^6^ identified expanding access to effective mental healthcare as a major challenge worldwide, including the transformation of health systems and policy responses.^7-9^ The World Health Organization (WHO) calls to promote mental well-being, prevent mental disorders, provide care, enhance recovery, promote human rights and reduce the mortality, morbidity and disability for persons with mental disorders.^3^

### Prevalence of Mental Disorders and Comorbidities in Peru

Data from 2012 show that 1 in 5 Peruvians are affected by a mental disorder.^10^ This pattern is not homogenous across groups; the economically impoverished and victims of political violence are disproportionally affected. Epidemiological studies conducted in different regions of Peru reveal that the annual prevalence of mental disorders is nearly twice as high among those who cannot cover their basic needs compared to those who can: 14.2%-41.8% versus 8%-15.8%, respectively.^11-14^ Ayacucho, the region most heavily affected by armed conflict in the 1980s and 1990s, has the highest lifetime prevalence of mental disorders relative to other regions of Peru, reaching 50.6% of the population.^15^ Given this scenario, it is not surprising that neuropsychiatric disorders are already the leading cause of disease burden in the country.^16^ In 2012, neuropsychiatric conditions accounted for the highest number of disability-adjusted life years (DALYs) lost with around 1 million years lost out of the total 5.8 million lost to all health conditions in Peru.^17^ In terms of individual diseases, depression ranked as the second leading cause of disease burden after respiratory tract infections in childhood.^17^

Despite their high prevalence and impact on treatment adherence for comorbid conditions, mental health disorders go largely undiagnosed and/or undertreated.^18,19^ For example, depression and chronic disease comorbidity is associated with reduced treatment adherence, poorer prognosis, greater disability, and higher mortality among sufferers of different physical diseases.^20^ In addition, among pregnant women, mental health disorders are associated with an underutilization of antenatal care services, premature birth, and lower birth weight.^21^ Traditionally, however, mental healthcare has not been delivered at the primary care level, where patients are most likely already receiving most of their routine care, but at the tertiary care level where mental health treatment can be delivered to only a small number of people, often the most seriously ill, and out of reach to the majority of persons needing assistance.

The primary healthcare setting provides the perfect venue to identify and treat most mental health disorders for a number of reasons. First, mental health disorders are often present simultaneously with other diseases. Again, using depression as an example, the concomitant prevalence of depression with other chronic diseases and pregnancy among primary healthcare attendees has been estimated at rates as high as 40% in pregnant women,^22,23^ 68% in women living with HIV/AIDS,^19^ 52% in patients with tuberculosis,^24^ and 30% in patients with diabetes.^25^ Second, these patients usually have frequent and long-lasting contact with first-line health professionals for regular check-ups and/or treatment, increasing the possibility to diagnose and treat their mental health conditions. This also accounts for patients without a chronic disease seeking care for a temporary physical health condition. In addition, the opportunity to diagnose mental health disorders at an early stage within primary healthcare settings can play an important factor in secondary and tertiary prevention of mental and physical health conditions.

### Mental Health Services Within the Peruvian Health System

Despite the implications of the high MNS disease burden, the great majority of people suffering from these disorders do not have adequate access to care. Results from epidemiological studies conducted by the Peruvian National Institute of Mental Health, at the forefront of identifying and measuring the mental healthcare gaps nationally, reveal the extent of the problem: among those, stating a need for mental healthcare, 69%-85% sought no care. The primary reasons conveyed for not seeking care were lack of financial recourses and information as to where to seek care.^11,12,14,26-28^

Deficiencies in the financial and human resources allocated to mental health^5,29^ explain, in part, the origins of the mental health service gap in Peru. The WHO’s Mental Health Atlas reports that, in 2011, the Peruvian Ministry of Health (MoH) allocated only 0.27% of its entire health budget to mental health, of which 98% went directly to psychiatric hospitals.^30^ Since most of the mental health budget was concentrated in specialized facilities, almost all of the mental healthcare services were available only at the tertiary care level. These deficiencies were not only observed in terms of budget allocation but also in the availability and distribution of human resources for mental health: in Peru, for a population of nearly 30 million people, in 2011, there were 1.71 psychologists and 0.57 psychiatrists per 100 000 inhabitants.^30^ These indicators lag far behind similar upper-middle income countries, where the median number of psychiatrists is 2.03 per 100 000 inhabitants.^31^ In 2014, of the 700 psychiatrists available in Peru,^32^ 85% were located in Lima, with half working in the private sector or in psychiatric hospitals.^10^ Of all the psychiatrists working for the MoH, only 20% work in general hospitals.^33^ This specialized and tertiary-level mental healthcare model implies significant shortages in other healthcare levels, thus, reducing the accessibility for the patients for diagnosis and treatment.

As reported in the Peruvian country profile of the Mental Health Atlas in 2011, primary care doctors and nurses could not prescribe psychotherapeutic medicines nor diagnose or treat mental disorders, and they had not received in-service training in mental health in the previous five years.^31^ Even today, additional observations arising from the Peruvian National Institute of Mental Health and other major stakeholders indicate an absence of the screening and diagnosis of mental health disorders using standardized protocols at the primary healthcare level; potential medication supply chain obstacles; limited capacity for mental health issues training for primary healthcare providers; and, limited efforts to expand to a community-wide mental health approach.

Taken together, from a health systems’ point of view, many of these challenges point towards identifiable bottlenecks that could be addressed at the macro level of policy and regulations, at the meso level of infrastructure and organization of services, as well as at the micro point-of-care level for both users and providers.

## Methods

In order to understand and consolidate the recent developments in mental health policy in Peru, we held several meetings with key individuals involved in the design and implementation of mental health reform in Peru to discuss details of the plans to implement the reform. The individuals we met with included key players in the reform process, such as the Peruvian MoH’s National Mental Health Coordinator, Dr. Yuri Cutipé, and the former Director of the Peruvian National Institute of Mental Health “Hideyo Noguchi,” Dr. Humberto Castillo, both of whom were directly involved in the architecture of the current mental health reforms in Peru. In addition, we reviewed the primary government health documents on which the reforms are based. The key government mental health documents were reviewed in order to describe the current state of the reform implementation. In doing so, we provide an understanding of the path that led to the beginning of the reform, how the ongoing reform is taking place, the plan and scope for scale-up, and the associated challenges for implementing such developments.

The documents reviewed were selected due to their relevance in moving the mental health agenda forward within the Peruvian public health system. First, the Guiding Principles for Action in Mental Health, published in 2004,^34^ laid the foundation for the creation of the *Estrategia Sanitaria Nacional de Salud Mental y Cultura de Paz* (National Health Strategy for Mental Health and Culture of Peace), a key agency of the MoH devoted to the supervision and monitoring of the implementation of mental health policies nationwide. Second, the National Plan for Mental Health of 2006^35^ established a 5-year action plan to strengthen the mental healthcare provided across the country. These efforts to improve mental health conditions made way for the passing of Law 29889,^36^ which started the current mental health reform process. Finally, the “Control and Prevention in Mental Health” (PpR 131)^37^ and The Ministry of Economy and Finance’s online public information consultation system^38^ were reviewed to better understand the current budget allocation for the mental health initiatives.

## Results and Discussion

### Recent Developments in Mental Health in Peru

In 2013, Peru began nationwide healthcare reform, with the objective of improving the quality and coverage of the healthcare system. Universal health coverage and the provision of health insurance for the poorest are the most salient features of this ongoing national reform.^39^ As with any major healthcare reform, and considering its major repercussions for mental health, it is necessary to map-out the major milestones achieved in the past in order to anticipate the strength and scope of current or planned changes.

In 2004, the MoH approved the Guiding Principles for Action in Mental Health.^34^ This document was designed to serve as a basis towards the development of a National Plan for Mental Health. The National Plan for Mental Health was approved in 2006,^35^ developed with recommendations from the World Health Report 2001 “Mental Health: New Understanding, New Hope.”^40^ To achieve those recommendations, Peru’s National Plan for Mental Health centered on four major objectives: (*i*) positioning mental health as a constitutional right; (*ii*) strengthening the role of the MoH in mental health activities; (*iii*) ensuring universal access to integral mental healthcare, beginning with the re-structuring of services to prioritize community-based mental healthcare; and (*iv*) promoting equity in mental healthcare, with an emphasis on gender, socioeconomic position, lifecycle, and cultural diversity. Of note, regionally, other Latin American countries, such as Argentina, Brazil, Chile, and Panama, have made steps towards implementing mental health policies and plans to differing degrees, leading to a rethinking of the hospital-based model and a restructuring of mental healthcare delivery to the primary care health centers as well as a community-based care component including community mental health centers.^41^ For example, Brazil, Chile, and Panama are the furthest in implementing their national mental health policies and plans and mental health hospital downsizing while Argentina has focused reform in only one province (Rio Negro).^42^ But while mental health legislation has been critical for the reforms in Brazil and Rio Negro, it has not been for Chile and Panama.

In June 2012, The Peruvian Congress, following the guiding principles of the Declaration of Caracas which set out to restructure psychiatric care in Latin American countries towards a community-based model,^41^ and demonstrating a commitment to implement the National Plan for Mental Health, passed landmark legislation, Law 29889, titled “[…] General Health Law guaranteeing the rights of people with mental health problems.”^36,43^ This Law explicitly guarantees the availability of programs and services for mental healthcare country-wide, including interventions related to the promotion, prevention, recovery and rehabilitation of every citizen at every level of the healthcare system, a substantial achievement for mental health in Peru.

It must be acknowledged that, though the political and legal framework driving mental health reform has taken more than a decade to establish, it would not have been possible without both the consistent and strong leadership from within the MoH but also strong political will that spanned three different Presidents since 2004. Moreover, beyond the WHO, other international actors strongly supported mental health reform in Peru as evidenced by the presence of the President of the World Bank at the signing of the directives for Law 29889 that established the framework for the new mental healthcare delivery model.^43^ The ultimate goal of Law 29889 was to transform mental health service delivery into a community-based healthcare model, setting it apart from most other countries in the region (with the notable exceptions discussed above), which still rely heavily on centralized service delivery models.^41^ The approach of the Peruvian mental health reform is to strengthen the role of the community in the treatment and rehabilitation of patients with mental disorders and increase access to mental healthcare. National and regional mental health authorities, particularly the Peruvian National Institute of Mental Health, are tasked to lead the implementation and scale-up of the reform at the primary care level country-wide. The activities implemented are based on recommendations by the WHO,^3^ and involve the task-shifting of detection and treatment of mild to moderate disorders from specialists to non-specialist health providers,^4^ the implementation of community-based mental health facilities that will reduce the burden on the few psychiatric hospitals available in Peru,^41^ and a restructuring of general hospitals to include beds for brief hospitalization and emergency treatment for patients with mental disorders which was previously unavailable within the Peruvian public health system.

The mental healthcare reform comprises the following pillars^43^:

(*i*) Restructuring of mental healthcare services at the primary and secondary care level: This pillar focuses on shifting the current mental healthcare delivery system by strengthening the role of primary health centers and general hospitals. This will help tackle one of the healthcare system’s major deficiencies: detection of mental health distress and disorders at the primary care level. As of June 2015, training sessions with primary healthcare providers from Lima and other regions such as Tumbes, Madre de Dios, and Loreto had been initiated. The training aims to incorporate into their care routines the detection of mental disorders and prescription of pharmacological treatment for mild to moderate disorders by general practitioners. In future years, trained providers are expected to be able to detect and treat most common mental disorders.

Patients with severe mental disorders are referred to Community Mental Health Centers (CMHCs) for treatment. These facilities are expected to become a key component in the mental health service delivery system in Peru by supporting the decentralization of mental healthcare from psychiatric hospitals to the community level. CMHCs staff include a psychiatrist and a team to provide specialized outpatient services for children, adolescents, adults and elderly patients with mental health disorders, psychosocial problems and addiction. A core activity of the CHMCs is to provide technical assistance to the primary care centers, support community integration, and liaise with other existing services. Once the mental health reform tasks are implemented, the CMHCs will also assume the responsibility of providing clinical support to the primary health centers from the Peruvian National Institute of Mental Health and the psychiatric hospitals. Currently, there are 29 CMHCs implemented in 6 regions of Peru. Emergency cases and patients who require hospitalization will be referred from the CMHCs to general hospitals in order to reduce the burden (including the number of users, number of admissions, number of beds occupied, and staff workload, etc) on psychiatric hospitals, and slowly begin to transfer these tasks to the general hospitals. Prior to the reform, hospitalization for psychiatric conditions was primarily available at psychiatric hospitals in Lima, and often lasted for an undetermined period of years, which as a result increased stigma associated with these hospitals and the mental health conditions they sought to treat as well as delaying the reintegration of stable patients back into society. Treating mental disorders like other health conditions seen at general hospitals is expected to reduce stigma and bring mental healthcare closer to the patients and their families. Once patients are discharged, they will be referred back to CMHCs to be included in a continuity of care program.

While the community-based model is strengthened and consolidated, and gains prominence within the community and the health system, psychiatric hospitals will be tasked with providing support and supervision for the implementation of mental health activities at the primary healthcare centers and general hospitals. The support component of the reform aims to provide primary health centers with the necessary skills to not only provide quality mental healthcare, but also to establish mental health tasks as part of their prioritized plans and to obtain and manage the resources necessary to carry out these activities for long term sustainability. The supervision component aims to monitor the implementation of the mental health tasks through the review of clinical records, budget expenditure reports and interviews with health center personnel. The reform will inevitably lead to a restructuration of the psychiatric hospitals in the near future, and their role will most likely become more similar to a general hospital with a psychiatric service, rather than serving as a psychiatric hospital only. In addition, one, the hospitals, the Peruvian National Institute of Mental Health, will focus on their academic role of promoting research and innovation in mental health.

(ii) *Creating supporting medical services to aid in the recovery and reintegration of patients to society:* Four new institutions will be created in order to provide a community-based mental healthcare network: (*a*) Protected homes or halfway houses for patients discharged from psychiatric hospitals lacking family support; (*b*) Protected residences for discharged patients who suffer from disabling sequelae and require additional care; (*c*) Specialized psychosocial rehabilitation centers to help patients recover their autonomy and provide support to families as patients reintegrate into society; and (*d*) Vocational rehabilitation centers which are designed to help patients recover or improve their job skills.

These new institutions promote a deinstitutionalization model and replace traditional hospital-based care with a community-based approach to treat mental disorders. These facilities will work closely with the CMHCs to complement and optimize patient recovery by covering basic needs and providing them with skills to facilitate their reintegration. The Peruvian National Institute of Mental Health is also charged with the task of increasing awareness about mental health conditions and services within other governmental and non-governmental entities, as well as authorities and the general population (eg, via the media) not only to improve the uptake of mental health services but also to secure and protect the rights of mental health patients.

(*iii*) *Supplying health centers with psychiatric medication*: By improving diagnosis, the demand for mental healthcare will rise, and more psychiatric medication will be required. Therefore, as part of the training conducted by the Peruvian National Institute of Mental Health, general practitioners at primary care centers are receiving training to prescribe standardized psychiatric medication for depression, anxiety, psychosis, and convulsive disorders. In addition, the general practitioners are responsible for designing treatment plans based on the clinical practice guides from the MoH, just as they do for non-mental health conditions requiring treatment.

(*iv*) *Expanding insurance coverage to include mental health: the Seguro Integral de Salud* (SIS, Integral Health Insurance) is a government-sponsored health insurance plan for the most vulnerable populations such as people living in poverty and the unemployed. SIS now covers mental healthcare services including ambulatory care and medication^44^ as outlined in an Executive Resolution which added mental health screening as part of the basic care package offered by primary health centers.^45^ Furthermore, a portion of health center budgets is tied to achieving this aim. In 2014, SIS added a “Percentage of patients screened for mental disorders” indicator.^46^ The Regional Health Directorates (*Direcciones de Salud* or DISAS), already in place with the objective of overseeing a group of health centers within the same geographical location, will perform quarterly performance evaluations of each indicator.^46^ Additionally, The *Superintendencia Nacional de Salud* (SuSalud), a government oversight entity whose mission is to protect the right to health for every Peruvian citizen, is in charge of monitoring the performance indicators based on the information registered in the SIS system (SIASIS). Consequently, health centers will need to include mental healthcare in their routine practices in order to access their complete budget. This is a major step in closing the treatment gap and guaranteeing universal access to mental healthcare at every level.

Financial resource allocation also demonstrates a commitment to mental health reform. A 10-year budget program, approved by the Ministry of Economy and Finance in 2014, accompanies the implementation and scalability of this normative framework.^47,48^ This budget program named “Control and Prevention in Mental Health,” (PpR 131), was allocated PEN 78 million (~US$20 million) in fiscal year 2015, and was used exclusively for mental healthcare reform activities (see Table for further detail).^47^ Unspent funds revert back to the Ministry of Economy and Finance, which then leads to a reassessment of the amount assigned and a possible reduction in the budget allocated for the next month. In this way, managers and providers are encouraged to achieve the proposed goals related to mental health tasks. In addition, the regulations of Law 29889 state that health institutions should allocate at least 10% of their budget for training and capacity building of their human resources in mental healthcare.^43^

Importantly, this reform provides a clear signal of shifting services and resources from psychiatric hospitals to community mental health facilities, together with the integration of mental healthcare services into primary care. Many of the initiatives currently undertaken by the MoH and its specialized mental health institutes are in motion. In the next section, we provide more detail about the plans, targets and budget to scale-up these activities to other regions of Peru in the following years.

### Imminence of Scale-up Actions in Peru: Matching Resources to Measurable Indicators

The mental healthcare reform scale-up would not be possible if it lacked resources and indicators to monitor progress. Similar to the SIS, the Peruvian Ministry of Economy and Finance assigns budgets based on the attainment of predetermined indicators, otherwise known as *Presupuesto por Resultado* (PpR) or pay-for-performance approach. The “Control and Prevention in Mental Health” program (PpR 131) is a 10-year program that has been approved and resources have been allocated to activities with measureable indicators.

The Ministry of Economy and Finance’s online public information consultation system shows budgets under implementation,^38^ confirming the governmental commitment. As stated above, the PpR 131 allocated PEN 78 million (~US$20 million) for fiscal year 2015 independent from other resources assigned to the health sector, that is, to be used exclusively for mental healthcare reform activities. This budget, summarized in Table, is attached to certain pre-defined indicators which are measurable at the primary care level.^37,48^ This is further proof of the imminence of the initiation and implementation of the reform.

**Table:** Funding Committed, Geographical Scope, Activities and Indicators for Mental Health, Fiscal Year 2015, as Per PpR 31^a^

	**Support and Supervision**	**Screening and Diagnosis**	**Treatment**	**Community Actions**
Budget ($US)	2 149 977	6 369 266	10 018 373	184 455
Purpose	Monitoring and evaluation of the implementation of the mental health program	Early detection of mental disorders	Opportune treatment for identified cases	Mental health promotion
Where	National, regional, and local			
Healthcare level	Primary, secondary, and tertiary	Primary	Primary, secondary, and tertiary	Primary and secondary
What	• Monitoring, supervision, evaluation and control of the mental health program • Creation of intervention guides for health workers • Epidemiological surveillance	Screening of: • Mental disorders (depression, anxiety, psychotic disorders, alcoholism) • Poor social skills in children and teenagers	Treatment for: • Depression and anxiety • Psychotic disorders • Alcoholism	• Community health workers and neighborhood councils trained to promote and improve mental health in their communities • Educational sessions for families • Community interventions for victims of political violence

^a^Information derived from Ministry of Economy and Finance’s online transparency portal.

In summary, this manuscript provides important information about the landmarks achieved, including legislation, budgets, and devising plans for implementation and monitoring of mental health reform in Peru. The fact that this manuscript is written before the reform is well underway is a strength because it provides a document that can be used for reference as the experiment proceeds. Next, we discuss some of the challenges for the implementation and scale-up of these efforts.

### Challenges for Implementation and Scale-up

While the commitment and will to provide financial resources to the implementation and scale-up of mental health reform has been demonstrated, these are insufficient to ensure successful incorporation of the new tasks, functions and responsibilities into existing care routines at the primary care facilities and general hospitals. Potential implementation knowledge gaps, and identified substantial obstacles to the scale-up efforts must be addressed in order to pave the way for a successful implementation of Peru’s ambitious but achievable goals in the arena of mental healthcare.

The first major obstacle is the context-specific barriers for the implementation of mental health initiatives. Mental health reform must be deployed nationwide to support screening, diagnosis and treatment efforts at the primary care level, which together account for ~87% of the program’s budget (~US$16.5 million, see Table).^38^ This deployment will occur through a health system with many weaknesses, especially prominent in isolated rural areas. While mental healthcare reform has secured many legal, financial, and operational means to become reality, we need to address how, at the ground level, the complexity between context and adaptation to new tasks, challenges, and opportunities can converge towards fostering further action to reduce mental health gaps rather than inducing inactivity. By doing so on an ongoing basis, in different regions, lessons will be learned, providing early warnings of many potential obstacles that need to be resolved on the path towards scaling-up mental health services.

A second major obstacle is the limited capacity of specialized mental health institutes to substantially grow towards securing the provision of training and clinical support activities at the same rate that the scaling-up efforts are taking place. The challenges of providing training and support will likely increase significantly as reform increasingly extends beyond the capital, Lima, towards the ultimate goal of reaching all regions of Peru. This is apparent from the financial and human resources challenges, with a current allocation of only US$2 million to cover 25 regions. Over time, those who govern not only the public health system but the country itself will change, and funding priorities often shift. It will be necessary for the current leaders to guide the next generation of leaders at all levels in the MoH, from governance to care delivery, in order to ensure that reform stays on track and does not become a failed experiment. To the extent possible, current leaders should continue to “hard code” reform into law and policy, and project a posture of transparency regarding the gains and setbacks to both the local and international communities as the initial data on its first year of reforms emerges.

In addition, policy-makers should consider that decentralization of mental healthcare into primary healthcare is only one component of a multi-level approach. At the heart of a community-based healthcare model is, of course, the community, and all of its facets: individuals, families, organizations, institutions, and so on. The Peruvian Government has laid the groundwork for the mental healthcare reform, but the ultimate success of the reform will rely heavily on the ability of these various community facets to join forces and contribute to the cause. The traditional mental health service system is a specialized, tertiary-level model that has proven insufficient in meeting the great burden of society’s mental health needs. Global mental health approaches, therefore, emphasize not only the decentralization and diffusion of care to the primary healthcare center level, but, optimally to household level, as well, though community-based initiatives. Many common mental health disorders can be identified and managed by trained laypeople (community health workers) in conjunction with outpatient community mental health centers and group homes or halfway houses as stable but chronically hospitalized individuals are reintegrated into society. Community-based, non-profit organizations, and other community organizations will play an increasingly important role in this area, ideally in close collaboration with governmental institutions so that a true mental health continuum of care can be established and maintained.

The Peruvian National Institute of Mental Health has acted progressively in this regard, by including from the beginning of its reform civil society members, non-governmental organization’s (NGO’s) and universities in much of the planning and early roll-out of services. NGO’s can help with patient identification, education and referral as well as the delivery of low-intensity non-pharmacological treatments for some common disorders, eg, mild depression, while universities can support efforts aimed at research activities that can measure, describe and support the implementation process in these crucial first few years. While no integrated system-wide plan for the monitoring and evaluation of the scale-up is currently in place (which would ideally include how patients/users do under these reforms), there is both great need and opportunity for work in this area while implementation is still in its infancy. For a scaled-up mental health reform to be successful, these key players along with community-based interventions need to be considered and included to reach an effective multi-level mental healthcare approach.

## Conclusion

Mental health service delivery has been prioritized by the Peruvian government, as demonstrated by a legal framework to standardize and regulate care at the primary level coupled with increased financial and human resources matched to measurable indicators. While the full impact of the mental healthcare reform is yet to be seen, the confluence of various legal and regulatory achievements places Peru on a path towards improving the quality of care provided at all levels of the health system and closing its mental health treatment gap.

## Acknowledgments

MT, VC, FDC, and JMM are supported by Grand Challenges Canada’s Global Mental Health initiative (GMH 0335-04). LRB, FDC, and JJM are supported by National Institute of Mental Health, National Institutes of Health, Department of Health and Human Services (1U19MH098780). The CRONICAS Centre of Excellence in Chronic Diseases at Universidad Peruana Cayetano Heredia was funded by the National Heart, Lung and Blood Institute, National Institute of Health, Department of Health and Human Services (project number No. HHSN268200900033C).

## Ethical issues

This review did not involve human subjects and did not require human ethics committee review.

## Competing interests

The authors declare they have no competing interests.

## Authors’ contributions

All authors contributed substantially in the search for information as well as the drafting of this review article.

## Authors’ affiliations

^1^CRONICAS Center of Excellence in Chronic Diseases, Universidad Peruana Cayetano Heredia, Lima, Peru. ^2^Instituto Nacional de Salud Mental “Honorio Delgado - Hideyo Noguchi,” Lima, Peru. ^3^Socios en Salud, Lima, Peru. ^4^Department of Global Health and Social Medicine, Harvard Medical School, Boston, MA, USA. ^5^Ministerio de Salud, Lima, Peru. ^6^School of Medicine, Universidad Peruana Cayetano Heredia, Lima, Peru.

## Key messages

**Implications for policy makers**

- The achievement of important legal and fiscal policy milestones provides a framework in which the mental health reform in Peru is taking place. Additionally, a specific budget allocation for the implementation of this reform guarantees a setting in which the changes are fostered and activities are implemented.
- Despite this, it is important to identify the implementation challenges to overcome, for example, obstacles to the medication supply chain or the limited capacity for mental health training for primary healthcare providers.
- These challenges have to be addressed at the macro, meso, and micro levels, and include policy-makers, infrastructure and organization of services, as well as the micro point-of-care level of users and providers.

**Implications for public**

The implementation of a mental health reform process creates fertile ground on which to grow and improve mental healthcare delivery. In doing so, governmental institutions will need to collaborate with community-based, non-profit organizations as well as academic organizations in order to develop innovative and efficient implementation approaches to tackle the challenges that arise from implementing the reform activities within the public health system.
